# Lifestyle and emotional well-being in men and women with type 2 diabetes (e-VitaDM-4; ZODIAC-48)

**DOI:** 10.1080/13814788.2017.1292348

**Published:** 2017-03-22

**Authors:** Steven H. Hendriks, Evelien G.W. van Soldt, Michael van Vugt, Klaas H. Groenier, Yvonne Roelofsen, Angela H. E. M. Maas, Henk J. G. Bilo, Nanne Kleefstra, Kornelis J. J. van Hateren

**Affiliations:** ^a^ Diabetes CentreIsala, ZwolleThe Netherlands; ^b^ Department of General Practice, University of Groningen and University Medical Centre GroningenGroningenThe Netherlands; ^c^ Department of Medical Psychology, VU University Medical CentreAmsterdamThe Netherlands; ^d^ Department of Cardiology, Radboud University Medical CentreNijmegenThe Netherlands; ^e^ Department of Internal Medicine, University of Groningen and University Medical Centre GroningenGroningenThe Netherlands; ^f^ Department of Internal Medicine, IsalaZwolleThe Netherlands; ^g^ Medical Research Group LangerhansZwolleThe Netherlands

**Keywords:** Diabetes, lifestyle factors, emotional well-being, gender

## Abstract

**Background:** Whether lifestyle is associated with well-being in patients with type 2 diabetes (T2D) is largely unknown. Uncovering and clarifying associations between these constructs may lead to new strategies for improving both.

**Objectives:** The aim was to investigate the relationship between lifestyle and well-being, focussing on gender differences.

**Methods:** This cross-sectional study included 1085 patients with T2D that participated in the e-Vita part of the Zwolle outpatient diabetes project integrating available care (ZODIAC) study. Patients were included from May 2012 until September 2014 from 52 general practices. Emotional well-being was assessed with the World Health Organization-5 well-being index (WHO-5). Lifestyle information on body mass index, smoking, physical activity and alcohol use was extracted from self-reported questionnaires. Multiple linear regression analyses were used.

**Results:** After adjustment for other lifestyle factors, physical activity, smoking and drinking 22–35 alcohol consumptions per week were associated with the WHO-5 score in men and physical activity and smoking were associated with the WHO-5 score in women. In the fully adjusted analyses for the total study population, physical activity and smoking were still associated with the WHO-5 score (b = 1.1, *P* < .001 and b =-3.1, *P* = .018, respectively). In the fully adjusted analyses stratified to gender only physical activity was associated with the WHO-5 score (in men: b =0.8, *P* = .006, in women: b = 1.4, *P* = .001).

**Conclusion:** This study shows a negative, non-clinically relevant association between smoking and emotional well-being in the total population with T2D and a positive, non-clinically relevant association between physical activity and emotional well-being in both men and women with T2D.

KEY MESSAGES

- In both men and women with type 2 diabetes, a higher level of physical activity is associated with a non-clinically relevant higher degree of emotional well-being.
- In the total population with type 2 diabetes, smoking is associated with a non-clinically relevant lower degree of emotional well-being.

## Introduction

Low emotional well-being is found to be a good predictor of depression in patients with type 2 diabetes mellitus (T2D), which affects one-third of the patients with T2D [1–3]. This is a disturbing fact, since T2D and depression strengthen each other’s health risks [4,5].

A major determining factor in the development and maintenance of both diabetes and depression is a lifestyle. In previous studies, associations between body mass index (BMI), inactivity, tobacco smoking, alcohol consumption and depression in patients with T2D are described [6–9]. However, it is mostly unclear whether these relationships can also be found for well-being in general in patients with T2D. Well-being can be defined as optimal psychological functioning. It is described as a dimension of mental health which acts independently from mental illness, although they are highly correlated [10]. Studies which have investigated the effect of physical activity on well-being showed contradictory results [11–14]. When associations between lifestyle factors and well-being could be found, this could lead to new strategies for improving both in patients with T2D. Eventually, this may lead to a better prevention of depression and better diabetes control.

There are important lifestyle differences between men and women. It is known that smoking rates are higher in men, and smoking in men is more closely related to food and alcohol consumption [15,16]. It is also suggested that women are more likely to be sedentary than men [17]. Furthermore, the prevalence of obesity is described to be higher among women than men with T2D [18] and it is also mentioned that obesity has an opposite association with mental health in women compared to men [19]. As gender differences in lifestyle and the association of lifestyle with mental health exist, we hypothesized that the association of lifestyle and well-being could also be different between men and women with T2D. Therefore, the aim of this study was to explore the relationship between different lifestyle components and emotional well-being with a special focus on gender differences.

## Methods

### Study population

**Figure 1.**
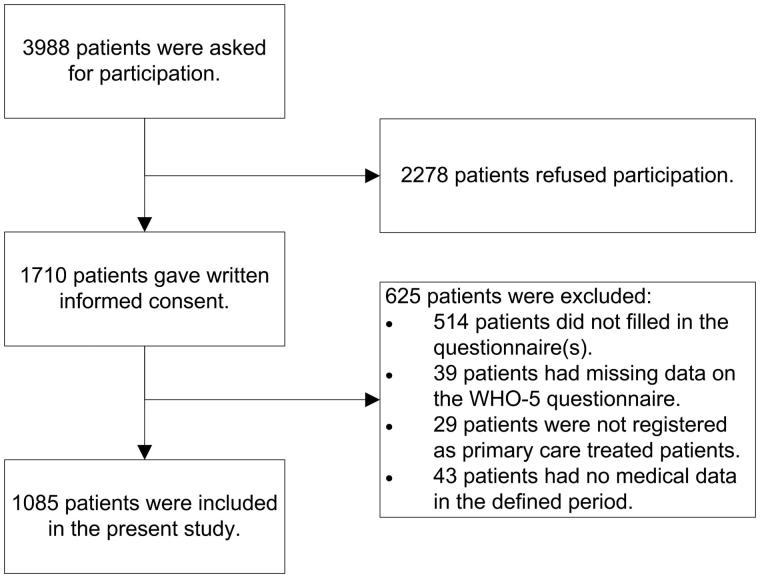
Flowchart of inclusion.

A cross-sectional analysis was performed using baseline data from the e-Vita part of the Zwolle outpatient diabetes project integrating available care (ZODIAC) study. This study is described in detail elsewhere [20]. The primary aim of the study was to investigate the effect of access to an online platform (e-Vita), meant to support and promote self-management, on the quality of life in patients with T2D treated in primary care. This study was conducted in general practices that are connected to the Care Group Drenthe in the Drenthe-region of the Netherlands (www.hzd.nu). Fifty-two out of the 110 general practices in this care group agreed to participate. In these practices, approximately 8300 patients with T2D were treated. Patients were recruited during a regular check-up by their practice nurse (PN) and were asked to fill out questionnaires including questions concerning lifestyle. Inclusion criteria for this study were age ≥18 years, a diagnosis of T2D and the general practitioner as main care provider for T2D. Patients were included from May 2012 until September 2014. A total of 1710 out of 3988 patients, who were asked to participate, gave written informed consent. The final study sample consisted of 1085 patients (13% of the total T2D population in the participating general practices); for more details see the flowchart in Figure 1.

### Emotional well-being

The World Health Organization-5 well-being index (WHO-5) was used to measure emotional well-being. This brief self-report test is recommended by the International Diabetes Federation as a screening tool for emotional well-being among patients with T2D [21].

The WHO-5 questionnaire contains descriptions of five different positive feelings: ‘I have felt cheerful and in good spirits,’ ‘I have felt calm and relaxed,’ I have felt active and vigorous,’ I woke up feeling fresh and rested’ and ‘my daily life has been filled with things that interest me.’ Subjects graded on a six-point Likert scale ranging from 0 (not present) to 5 (constantly present) how often each feeling was present in the last two weeks. A total score was calculated in which the maximum score was 100 (best possible well-being) [2].

### Lifestyle factors

The following lifestyle factors were included in this study: BMI, alcohol use, smoking and physical activity. Information on alcohol consumption was derived from the questionnaire at baseline. Patients had to choose from the following categories: no alcohol, seven glasses or less, 8–21 glasses, 22–35, or more than 35 glasses per week. Information on smoking and physical activity was derived from the summary of diabetes self-care activities measure (SDSCA) [22]. This self-report questionnaire covers behaviour in the week before filling out the questionnaire. Smoking status was categorized in non-smokers and current smokers. Physical activity was defined as the amount of days per week of at least 30 minutes physical activity.

### Other variables and data sources

Baseline demographic data included sex, age, education level and occupation. Educational status was categorized as low, intermediate or high. Occupation was categorized into four groups; having a job (full-time or part-time), being unemployed or incapacitated, being retired or being a homemaker. Medical data were extracted from the diabetes-specific database at our diabetes centre. This centre gathers data of primary care treated patients with T2D in a large part of the Netherlands on a yearly basis, to provide benchmark information to general practitioners. This database includes data on physical examination, use of medication, and laboratory blood and urine tests.

The following data were extracted: duration of diabetes, BMI, systolic blood pressure, HbA_1c,_ the presence of macrovascular complications (MVC) and information on (indicators of) diabetes-related complications, such as diabetic retinopathy (DRP), albuminuria and diabetic peripheral neuropathy (DPN). MVC was defined as (a history of) angina pectoris, myocardial infarction, percutaneous transluminal coronary angioplasty, coronary artery bypass grafting, stroke or transient ischaemic attack. Microalbuminuria was defined as an albumin-creatinine ratio between 2.5–25 mg/mmol in men and 3.5–35 mg/mmol in women. Macro-albuminuria was defined as a ratio higher than 25 mg/mmol and 35 mg/mmol for men and women, respectively [21]. An ophthalmologist determined presence of DRP once in two years. Foot sensibility was tested with 5.07 Semmes-Weinstein monofilaments. DPN was defined as two or more errors in a test of three, at least at one foot. Glucose-lowering therapy was categorized into; dietary measures only, oral blood glucose lowering therapy and insulin therapy (with or without oral therapy).

### Statistical analyses

Analysis was performed using SPSS version 20.0. Before analysing baseline characteristics, multiple imputation was applied to impute missing values of the independent variables, assuming that missing data was random (MAR) or completely random (MCAR).

The relationship between lifestyle and emotional well-being was examined by linear regression, in which the WHO-5 score was used as the dependent continuous variable. Three different predefined regression models were examined. Relationships between WHO-5 score and lifestyle components; BMI, exercise, smoking and alcohol consumption were analysed in the first model. The second model adjusted for demographic factors; sex, age, level of education and occupation. Potential medical confounders were added in the third model; HbA_1c_, the presence of complications (DRP, MVC, microalbuminuria or DPN), and use of antidepressants. Age, HbA_1c_, BMI and physical activity were treated as continuous data. In addition to the regression analyses for the total cohort, analyses were repeated stratified according to sex. The semi-partial correlation coefficient (r_part_) was used to measure the correlation between the independent variables and well-being, while controlling that independent variable for the other independent variables. The degree to which the different models determined the WHO-5 score was evaluated by the explained variance, shown as adjusted R^2^.

### Ethics statement

This study was approved by the Medical Ethical Committee of Isala, Zwolle, the Netherlands. Registered on clinicaltrials.gov, study ID NCT01570140.

## Results

Baseline characteristics of the study sample are depicted in Table 1. Fifty-five per cent of the patients were male. Mean age was 66 (SD: 10) years in men and 65 (SD: 10) years in women.

**Table 1.** Baseline characteristics of the total population and stratified according to gender.

	Total	Men	Women
*n* [%]	1085 [100]	598 [55]	487 [45]
Age, (years)	65 (10)	66 (10)	65 (10)
Occupation			
* *Job	293 [27]	174 [29]	119 [24]
* *Unemployed/incapacitated	93 [9]	55 [9]	38 [8]
* *Homemaker	132 [12]	11 [2]	121 [25]
* *Retired	567 [52]	358 [60]	209 [43]
Level of education			
* *Low	370 [34]	178 [30]	192 [39]
* *Mediate	478 [44]	253 [42]	225 [46]
* *High	237 [22]	167 [28]	70 [14]
Clinical values			
* *Diabetes duration (years)^a^	7 (3–10)	7 (3–10)	7 (3–11)
* *BMI (kg/m^2^)	29 (26–32)	29 (26–32)	30 (27–33)
* *HbA1c (mmol/mol)	49 (44–54)	49 (44–54)	49 (44–53)
WHO-5 score	76 (64–80)	76 (68–84)	72 (60–80)
Vascular complications			
* *MVC	328 [30]	220 [37]	108 [22]
* *DRP	49 [5]	30 [5]	19 [4]
* *Albuminuria	145 [13]	119 [20]	27 [6]
* *DPN	224 [21]	134 [22]	90 [19]
Use of antidepressants	55 [5]	23 [4]	32 [7]
Hyperglycaemic therapy			
* *Diet	207 [19]	106 [18]	101 [21]
* *Oral therapy only	725 [67]	412 [69]	313 [64]
* *Insulin therapy	153 [14]	80 [13]	73 [15]
Smoking	199 [18]	104 [17]	95 [20]
Alcohol consumption^b^			
* *No alcohol consumption	509 [47]	203 [34]	306 [63]
* *Alcohol 1–7	423 [39]	272 [46]	151 [31]
* *Alcohol 8–21	145 [13]	116 [19]	29 [6]
* *Alcohol 22–35	8 [1]	7 [1]	1 [0]
Physical activity^c^	5 (3–7)	6 (3–7)	5 (3–7)

Values are values are depicted as number [%], means (SD) or median (25–75 percentiles).

BMI, body mass index; WHO-5, World Health Organization-5 well-being index: MVC, macrovascular complications; DRP: diabetic retinopathy; DPN: diabetic peripheral neuropathy.

*n* (%) of imputed values: level of education 13 (1.1), occupation 9 (0.8), BMI 14 (1.2), HbA1_c_ 5 (0.4), DRP 60 (5.2), albuminuria 54 (4.7), DPN 61 (5.3), smoking status 3 (0.3), alcohol usage 9 (0.8), exercise 7 (0.6).

^a^ Information missing in 14 subjects with normal emotional well-being.

^b^ Alcohol units/week.

^c^ Number of days a week, at least 30 minutes exercise.

The median score on the WHO-5 questionnaire was 76 (68–84) in men and 72 (60–80) in women.

### Lifestyle and emotional well-being

The results of the linear regression analyses are presented in Table 2. The first model, including only lifestyle factors, showed an association between BMI, physical activity, smoking, alcohol consumption and WHO-5 score. Only physical activity and smoking remained significantly related when adjusted for socio-economic and medical confounders (b = 1.1, *P* <.001, r_part_ = 0.13 and b = –3.1, *P* .018, r_part_ = –0.07) (model 3). Lower scores for emotional well-being were found in patients that are female, unemployed or incapacitated, having high levels of education, using antidepressants or having MVC or DPN.

**Table 2.** Multiple regression analysis for lifestyle factors and emotional well-being in total population (*n* = 1085 patients with type 2 diabetes).

	Model 1 adjusted R^2^ = .046			Model 2 adjusted R^2^ = .113			Model 3 adjusted R^2^ =.132		
	b (95%CI)	*P*-value	r_part_	b (95%CI)	*P*-value	r_part_	b (95%CI)	*P*-value	r_part_
BMI (kg/m^2^)	−0.2 (−0.4, −0.1)	.016	−0.07	−0.2 (−0.4, 0.0)	.136	−0.04	−0.1 (−0.3, 0.1)	.207	−0.04
Physical activity^a^	1.2 (0.7, 1.6)	<.001	0.14	1.1 (0.7, 1.6)	<.001	0.14	1.1 (0.6, 1.5)	<.001	0.13
Smoking	−4.4 (−7.0, −1.9)	.001	−0.10	−3.8 (−6.3, −1.2)	.003	−0.08	−3.1 (−5.6, −0.5)	.018	−0.07
No alcohol consumption^b^									
Alcohol 1–7^c^	2.9 (0.7, 5.1)	.009	0.08	1.5 (−0.6, 3.7)	.165	0.04	1.2 (−1.1, 3.1)	.271	0.03
Alcohol 8–21^c^	4.0 (0.9, 7.1)	.010	0.08	2.1 (−1.2, 5.0)	.180	0.04	1.3 (−1.7, 4.4)	.421	0.02
Alcohol 22–35^c^	−5.3 (−17.0, 6.4)	.374	−0.03	−3.5 (−14.8, 7.9)	.552	−0.02	−3.7 (−15.0, 7.5)	.517	−0.02
Gender (men–women)				−5.3 (−7.4, −3.1)	<.001	−0.14	−6.0 (−8.2, −3.8)	<.001	−0.15
Age (years)				0.0 (−0.1, 0.1)	.979	<0.01	0.1 (−0.1, 0.2)	.467	0.02
Job^b^									
Unemployed/incapacitated				−11.6 (−15.4, −7.8)	<.001	−0.17	−10.3 (−14.1, −6.5)	<.001	−0.15
Homemaker				−3.5 (−7.1, 0.2)	.063	−0.05	−2.8 (−6.4, 0.8)	.128	−0.04
Retired				0.6 (−2.5, 3.7)	.721	0.01	0.8 (−2.3, 3.8)	.612	0.01
Low level of education^b^									
Mediate level of education				−2.4 (−4.6, −0.2)	.036	−0.06	−2.1 (−4.3, 0.1)	.065	−0.05
High level of education				−4.4 (−7.2, −1.6)	.002	−0.09	−4.1 (−6.9, −1.4)	.003	−0.08
MVC							−2.7 (−4.9, −0.6)	.013	−0.07
HbA1c (mmol/mol)							0.0 (−0.1, 0.1)	.788	−0.01
Use of antidepressants							−8.0 (−12.4, −3.6)	<.001	−0.10
Albuminuria^d^							−1.3 (−4.3, 1.8)	.416	−0.02
DRP							−0.6 (−5.3, 4.2)	.813	−0.01
DPN							−3.8 (−6.4, −1.2)	.004	−0.09

BMI: body mass index; MVC: macrovascular complications; DRP: diabetic retinopathy; DPN: diabetic peripheral neuropathy; b: unstandardized regression coefficients; 95%CI, 95% confidence interval; r_part_: semi-partial correlation coefficient.

^a^ Number of days a week, at least 30 minutes exercise.

^b^ Reference category.

^c^ Alcohol consumption (*n*/week).

^d^ Albumin-creatinine ratio above 2.5 g/mol in men and 3.5 g/mol in women.

### Differences between men and women

In men, physical activity, smoking and drinking 22 to 35 alcohol consumptions per week were associated with the WHO-5 score after adjustment for other lifestyle factors (b = 1.1, *P* <.001, b = –3.2, *P* = .048 and b = –12.9, *P* = .025, respectively) (Table 3). In women, physical activity and smoking were associated with the WHO-5 score after adjustment for other lifestyle factors (b = 1.2, *P* = .003, and b = –5.6, *P* = .009, respectively) (Table 4). After adjustment for sociodemographic and medical confounders, only physical activity remained significantly associated with emotional well-being in both men and women (Tables 3 and 4).

**Table 3.** Multiple regression analysis for lifestyle factors and emotional well-being in men with type 2 diabetes.

	Model 1 adjusted R^2^ = .36			Model 2 adjusted R^2^ = .119			Model 3 adjusted R^2^ = .150		
	b (95%CI)	*P*-value	r_part_	b (95%CI)	*P*-value	r_part_	b (95%CI)	*P*-value	r_part_
BMI (kg/m^2^)	−0.1 (−0.3, 0.3)	.873	−0.01	0.0 (−0.3, 0.3)	.929	<0.01	0.1 (−0.2, 0.3)	.707	0.01
Physical activity^a^	1.1 (0.5, 1.7)	<.001	0.15	1.0 (0.5, 1.5)	<.001	0.14	0.8 (0.2, 1.3)	.006	0.10
Currently smoking	−3.2 (−6.3, 0.0)	.048	−0.08	−2.1 (−5.1, 0.9)	.174	−0.05	−1.7 (−4.8, 1.3)	.262	−0.04
No alcohol^b^									
Alcohol 1–7^c^	0.7 (−2.0, 3.4)	.617	0.02	0.1 (−2.5, 2.7)	.956	<0.01	−0.8 (−3.4, 1.8)	.568	−0.02
Alcohol 8–21^c^	1.5 (−1.9, 4.9)	.383	0.04	1.5 (−1.8, 4.8)	.368	0.04	0.2 (−3.2, 3.4)	.944	<0.01
Alcohol 22–35^c^	−12.9 (−24.2, −1.6)	.025	−0.09	−7.4 (−18.3, 3.4)	.180	−0.05	−8.7 (−19.4, 2.0)	.111	−0.06
Age (years)				0.0 (−0.2, 0.2)	.995	<0.01	0.1 (−0.1, 0.3)	.374	0.03
Job^b^									
Unemployed/incapacitated				−13.2 (−17.5, −8.8)	<.001	−0.23	−12.0 (−16.3, −7.6)	<.001	−0.20
Homemaker				−2.3 (−10.9, 6.9)	.609	−0.02	−2.4 (−11.1, 6.2)	.579	−0.02
Retired				2.4 (−1.2, 5.9)	.199	0.05	2.3 (−1.2, 5.9)	.195	0.05
Low level of education^b^									
Mediate level of education				−2.1 (−4.9, 0.6)	.133	−0.06	−2.2 (−5.0, 0.5)	.107	−0.06
High level of education				−3.4 (−6.5, −0.4)	.029	−0.08	−3.4 (−6.5, −0.4)	.029	−0.08
MVC							−3.2 (−5.6, −0.8)	.010	−0.10
HbA1c (mmol/mol)							−0.1 (−0.2, 0.0)	.106	−0.06
Use of antidepressants							−7.7 (−13.6, −1.8)	<.001	−0.10
Albuminuria^d^							−2.5 (−5.6, 0.5)	.105	−0.06
DRP							−1.8 (−7.0, 3.5)	.512	−0.03
DPN							−3.4 (−6.2, −0.6)	.018	−0.09

BMI: body mass index; MVC: macrovascular complications; DRP: diabetic retinopathy; DPN: diabetic peripheral neuropathy; b: unstandardized regression coefficients; 95%CI, 95% confidence interval; r_part_: semi-partial correlation coefficient.

^a^ Number of days a week, at least 30 minutes exercise.

^b^ Reference category.

^c^ Alcohol usage (*n*/week).

^d^ Albumin-creatinine ratio above 2.5 g/mol.

**Table 4.** Multivariate regression analysis for lifestyle factors and emotional well-being in women with type 2 diabetes.

	Model 1 adjusted R^2^ = .036			Model 2 adjusted R^2^ = .053			Model 3 adjusted R^2^ = .063		
	b (95%CI)	*P*-value	r_part_	b (95%CI)	*P*-value	r_part_	b (95%CI)	*P*-value	r_part_
BMI (kg/m^2^)	−0.2 (−0.6, 0.0)	.054	−0.09	−0.3 (−0.6, 0.1)	.075	−0.08	−0.2 (−0.5, 0.1)	.156	−0.06
Physical activity^a^	1.2 (0.4, 2.0)	.003	0.13	1.3 (0.5, 2.1)	.001	0.14	1.4 (0.6, 2.2)	.001	0.015
Currently smoking	−5.6 (−9.7, −1.4)	.009	−0.12	−5.8 (−10.0, −1.5)	.008	−0.12	−4.2 (−8.5, 0.2)	.057	−0.08
No alcohol^b^									
Alcohol 1–7^c^	2.5 (−1.2, 6.1)	.183	0.06	3.0 (−0.6, 6.6)	.105	0.07	3.5 (−0.2, 7.1)	.068	0.08
Alcohol 8–21^c^	1.5 (−5.5, 8.5)	.675	0.02	2.0 (−5.1, 9.1)	.584	0.02	2.0 (−5.1, 9.1)	.592	0.02
Age (years)				0.0 (−0.2, 0.2)	.901	−0.01	0.0 (−0.2, 0.2)	.897	0.01
Job^b^									
Unemployed/incapacitated				−9.5 (−16.2, −2.7)	.006	−0.12	−8.1 (−14.8, −1.3)	.020	−0.07
Homemaker				−4.7 (−9.8, 0.3)	.068	−0.08	−3.9 (−9.0, 1.2)	.132	−0.10
Retired				−1.7 (−7.3, 3.7)	.530	−0.03	−1.5 (−7.0, 4.0)	.632	−0.02
Low level of education^b^									
Mediate level of education				−2.9 (−6.6, 0.9)	.132	−0.07	−2.2 (−6.0, 1.6)	.279	−0.05
High level of education				−6.8 (−12.1, −1.5)	.012	−0.11	−6.4 (−11.6, −1.2)	.019	−0.10
MVC							−2.6 (−6.6, 1.5)	.254	−0.05
HbA1c (mmol/mol)							0.1 (−0.1, 0.3)	.505	0.03
Use of antidepressants							−7.9 (−14.6, −1.1)	.021	−0.10
Albuminuria^d^							1.6 (−5.8, 8.9)	.696	0.02
DRP							2.4 (−6.2, 11.0)	.593	0.02
DPN							−4.7 (−9.1, −0.3)	.037	−0.10

BMI: body mass index; MVC, macrovascular complications; DRP: diabetic retinopathy; DPN: diabetic peripheral neuropathy; b: unstandardized regression coefficients; 95%CI, 95% confidence interval; r_part_: semi-partial correlation coefficient.

^a^ Number of days a week, at least 30 minutes exercise.

^b^ Reference category.

^c^ Alcohol usage (*n*/week).

^d^ Albumin-creatinine ratio above 3.5 g/mol.

The explained variance of model 3 was higher in men, compared to women (R^2^ = .150 versus R^2^ = .063, respectively). Being unemployed or incapacitated, having a high level of education, using antidepressants and having DPN resulted in a lower WHO-5 score in both men and women. The WHO-5 score was also negatively associated with having MCV in men only.

## Discussion

### Main findings

The results of this study indicate that there is a positive relationship between physical activity and emotional well-being, in both women and men. Furthermore, a negative association between smoking and emotional well-being in the total population was found. Other variables that proved to be associated with a lower emotional well-being were being female, being unemployed or incapacitated, and having DPN or MVC. Although these associations were statistically significant, the strength of the relations is rather weak.

### Comparison with other studies

Studies concerning the effect of physical activity on well-being in patients with T2D, show contradictive results. Three studies among patients with T2D found a positive effect of exercise on well-being [11–13]. Alternatively, a study among 218 patients with T2D did not observe any change in well-being after participating in an exercise programme for 22 weeks [14]. None of these studies indicated a pre-defined clinical relevant difference. In the present study, no physical activity versus seven days of physical activity was associated with a difference of almost eight points on the WHO-5 questionnaire. Since a difference of 10% in WHO-5 is mentioned as a significant change, the association between physical activity and well-being is possibly only relevant for patients who have a low level of emotional well-being [23]. The limited clinical relevance of the association is also displayed by the low semipartial correlation coefficient for physical activity (r_part_: 0.12).

In the general population, a lower emotional well-being was found in smokers compared to non-smokers [24]. However, the clinical relevance of this difference was not reported. In the present study, smoking resulted in a score three points lower on the WHO-5 questionnaire, which could not be indicated as a clinically relevant difference. Furthermore, the semi-partial correlation coefficient for smoking was very low (r_part_: -0.07).

No statistically significant association between emotional well-being and BMI was found in our study, which is partly in line with earlier research findings concerning BMI and depressive symptoms in patients with T2D [8].

Studies concerning alcohol consumption and well-being in particular, have only been performed in the general population with contradictive results [25,26]. One study found a modest positive association of alcohol consumption with poorer psychological well-being [25], whereas another study found that moderate alcohol consumption in older adults is associated with a higher well-being compared to abstinence [26]. The absence of an association in our cohort could be the result of the use of alcohol categories instead of the exact number of alcohol consumptions which lead to data loss.

### Gender differences

Our hypothesis concerning differences between men and women in the association between lifestyle factors and well-being could not be confirmed in our study. Furthermore, except for a statistically significant effect of macrovascular complications in men only, no gender differences in associations between demographic or diabetes-related factors and emotional well-being were found. Nevertheless, a higher explained variance in the final model was observed for men compared to women. This seems to be mainly caused by a smaller effect of demographic factors on emotional well-being in women compared to men. Although no significant different between men and women was found, this may be related to a higher impact of unemployment or being incapacitated on well-being in men compared to women. The explained variance of the final model for women compared to men also suggests that emotional well-being in women appears to be stronger related to other, not included (possibly women-specific), factors.

### Strengths and limitations

The innovative aspects of this study are the focus on well-being and gender differences in a primary cared treated diabetes population in particular.

Some limitations of our study need to be mentioned. Since the design of this study was cross-sectional, no statement could be made about the underlying mechanisms of the observed relationships. Furthermore, only 27% of all patients who were asked to participate in the observational cohort study were included in the present study. Some selection bias might have occurred as participants were more often men and younger compared to the total diabetes population in the Drenthe region of the Netherlands. This could be an explanation for the low number of subjects with a potential depression found in our cohort as well. About the assessment of lifestyle, this study used self-reported questionnaires. Therefore, the severity of poor lifestyle habits may be underestimated, because socially desirable answers may have been given. The most important relationship was found for the level of physical activity. Even though information on intensity and type of activity remained unknown in our analyses, the Dutch guideline for healthy physical activity is nonetheless taken into account [27]. This guideline advises exercising 30 minutes a day for at least five days a week to maintain good health.

### Implications for clinical practice and further studies

The clinical relevance of the association between physical activity and well-being seems to be low. This might indicate that it is not effective to improve well-being by physical activity or vice versa. However, more prospective longitudinal intervention studies are needed to investigate this hypothesis.

## Conclusion

The study indicates that there is a positive, non-clinically relevant relationship between physical activity and emotional well-being in both men and women. Furthermore, a negative, non-clinically relevant relationship with smoking was found in the total population with type 2 diabetes.
